# Crustacean Protein Kinases A and C: Bioinformatic Characterization in Decapods and Other Non-Model Organisms

**DOI:** 10.3390/ijms262110585

**Published:** 2025-10-30

**Authors:** Talia B. Head, Jorge L. Pérez-Moreno, Laura E. Antizzo, David S. Durica, Donald L. Mykles

**Affiliations:** 1 Department of Biology, Colorado State University, Fort Collins, CO 80521, USA; jperezmoreno@umass.edu (J.L.P.-M.);; 2 Department of Biology, University of Massachusetts Amherst, Amherst, MA 01003, USA; 3 School of Biological Sciences, University of Oklahoma, Norman, OK 73019, USA; ddurica@ou.edu; 4 Bodega Marine Laboratory, University of California, Davis, Bodega Bay, CA 94923, USA

**Keywords:** crustacea, cAMP-dependent protein kinase, protein kinase A (PKA), protein kinase C (PKC), Y-organ, molting, decapod, ecdysteroid

## Abstract

The AGC kinases constitute a large and ancient gene superfamily with origins that coincided with the appearance of multicellularity. Three AGC kinase families—protein kinase A (PKA), protein kinase G (PKG), and protein kinase C (PKC)—mediate the actions of neuropeptide hormones, biogenic amines, and other ligands on various physiological processes in metazoans. Metazoans express two PKG types. Jawed vertebrates express three PKA catalytic (C) subunits, four regulatory (R) subunits, and twelve PKCs, organized into conventional, novel delta-like, novel epsilon-like, atypical, and protein kinase N (PKN) subfamilies. By contrast, invertebrate PKA and PKC sequences are not well characterized. Consequently, limited database resources can result in misidentification or mischaracterization of proteins and can lead to misinterpretation of experimental data. A broad phylogenetic and sequence analysis of CrusTome transcriptome and GenBank databases was used to characterize 640 PKA-C sequences, 1122 PKA-R sequences, and 1844 PKC sequences distributed among the Annelida, Arthropoda, Chordata, Cnidaria, Nematoda, Mollusca, Echinodermata, Porifera, Platyhelminthes, and Tardigrada. Phylogenetic analysis and multiple sequence alignments revealed conservation of certain PKA-C, PKA-R and PKC isoforms across metazoans, as well as diversification of additional taxon-specific isoforms. Decapods expressed four PKA-C isoforms, designated PKA-C_1_, -C_D1_, -C_GLY1_, and -C_GLY2_; five PKA-R isoforms, designated PKA-RI_1_, -RI_D1_, -RII_GLY_, and -RII_D1_; and five PKC isoforms, designated PKN_D1-3_, conventional cPKC_D1_, novel nPKC_D1δ_ and nPKC_D1ε_, and atypical aPKC_D1_. PKA-C_GLY1_, -C_GLY2_, and -RII_GLY_ had glycine-rich N-terminal sequences that were unique to crustaceans. These data suggest lineage-specific diversification that retained the core catalytic function of each kinase, while regions outside of the kinase domain may provide specialized regulatory mechanisms and/or spatiotemporal subcellular localization in invertebrate tissues.

## 1. Introduction

The AGC kinase gene superfamily is composed of 63 serine/threonine protein kinases, distributed among 14 families [1,2]. Among these are cAMP-dependent protein kinase (PKA), cGMP-dependent protein kinase (PKG), and protein kinase C (PKC) families, after which the superfamily is named. AGCs are downstream effectors of a broad range of signals, including growth factors and synaptic transmission, among others [2,3]. AGC kinases share a common catalytic domain as well as conserved features such as an activation loop located in the kinase domain and a hydrophobic motif located C-terminal to the kinase domain [1,2,4]. Where the AGC kinases have diverged is in the mechanisms of regulation, which can include cyclic nucleotides, calcium, phospholipids, and phosphorylation, including autophosphorylation, to name a few [1,2,4,5].

PKA is a tetramer composed of two catalytic (PKA-C) subunits, each bound to two regulatory (PKA-R) subunits [6,7]. In vertebrates, two genes encode two different PKA-C subunits, alpha (α) and beta (β), as well as a third gene encoding the gamma (γ) subunit that is only found in primates, and four genes encode four different PKA-R subunits, RIα, RIβ, RIIα, and RIIβ [8,9,10]. PKA-R consists of an N-terminal dimerization and docking (D/D) domain consisting of a coiled-coil motif, followed by two cAMP-binding domains. PKA-R contains a pseudosubstrate motif that binds to the catalytic domain, suppressing catalytic activity. The D/D domain allows for dimerization of the C_1_R_1_ dimer to form the inactive PKA tetramer [1,4,11,12]. The D/D domain also interacts with A-kinase anchoring proteins (AKAPs) to direct the holoenzyme to discrete intracellular locations [9,10,13,14,15]. PKA activation requires phosphorylation, typically autophosphorylation, of Thr197 in the activation loop of PKA-C, as well as cAMP binding to PKA-R (reviewed in [12]). Activation loop phosphorylation and binding of cAMP induce a conformational change and the dissociation of the regulatory and catalytic subunits [6,11,12]. Phosphorylation outside of the activation loop plays a regulatory role, including autophosphorylation of the pseudosubstrate in regulatory subunits. In PKA-RII, autophosphorylation of the pseudosubstrate motif reduces binding affinity to PKA-C and the overall activation threshold of PKA, whereas the pseudosubstrate of PKA-RI lacks a phosphorylatable residue [6,15,16].

PKC is a single polypeptide with an N-terminal regulatory region and a C-terminal catalytic region. There are five main families of PKCs, with 12 isoforms characterized in vertebrate species: conventional (α, β, and γ), novel delta-like (delta [δ] and theta [θ]), novel epsilon-like (epsilon [ε] and eta [η]), atypical (iota [ι] and zeta [ζ]), and three protein kinase Ns (PKN1-3, also called PKC-like) [2,17]. PKCs are distinguished by differing combinations of conserved domains: conserved region 1 (C1), conserved region 2 (C2), homology region 1 (HR1), Phox and Bem1 (PB1), pleckstrin homology (PH), and the catalytic domain [2,17]. C1 is a membrane-targeting region that binds diacylglycerol (DAG) or phorbol esters [18,19]. Following ligand binding, the hydrophobic surface of the C1 domain enables interaction with membrane phospholipids [19]. Calcium binding to the C2 domain induces a conformational change that increases enzyme affinity for DAG and membrane phospholipids [20,21]. PB1 mediates specific protein–protein interactions with other PB1-domain-containing proteins [22,23]. The HR1 domain binds Rho family GTPases, which activate PKNs as opposed to DAG and/or Ca^2+^ seen in other PKCs [24,25,26].

PKA, PKC, and PKG signaling pathways are involved in every aspect of crustacean biology, as reviewed in [27,28]. For example, molt-inhibiting hormone (MIH) signaling in the crustacean molting gland, or Y-organ (YO), links a PKA-dependent triggering phase to a PKG-dependent summation phase to inhibit synthesis of molting hormones (ecdysteroids), as reviewed in [28,29,30]. Whether specific PKA isoforms are involved in MIH signaling is unknown.

MIH activates PKG1 and PKG2, which have opposite effects on ecdysteroid secretion: PKG1 inhibits secretion, whereas PKG2 partially counters PKG1 by stimulating secretion [31]. Activation of PKC stimulates YO ecdysteroid secretion via a pathway distinct from MIH, as reviewed in [29,32]. Whether multiple PKC isoforms are involved has not been investigated. Gonad/vitellogenesis-inhibiting hormone (GIH/VIH) directly inhibits vitellogenesis and ovarian maturation via PKA, PKC, or PKG signaling [33] and is reviewed in [27,34,35,36]. These examples show that crustaceans integrate a diverse array of hormones and GPCRs that elicit appropriate responses in target tissues via PKA-, PKC-, or PKG-dependent signaling.

Although PKA and PKC play important roles in crustacean physiology, their molecular identities and mechanisms of action are poorly understood. In particular, little is known about the domain organization, the conservation of catalytic motifs, and the phylogenetic origins and diversity of crustacean PKA and PKC homologs. Comparative genomic analyses of PKA and PKC genes have emphasized chordate taxa, with limited representation from a few non-chordate taxa [2,17,37]. To better understand how these kinases have evolved to support lineage-specific physiology, such as molting, it is necessary to first characterize and classify their foundational sequences and structures. In this study, we identified and characterized PKA and PKC homologs among decapods, using the same bioinformatic and phylogenetic approaches that were applied to metazoan PKGs [31]. Taking advantage of a crustacean transcriptomic database, CrusTome [38], we characterized PKA and PKC sequences across metazoans, with an emphasis on pancrustacean taxa. The results indicate that the origins of PKA and PKC predate multicellularity and that these kinases have undergone lineage-specific diversification that may indicate specializations associated with life history traits.

## 2. Results

Phylogenetic analysis of pancrustacean and tardigrade sequences from CrusTome, along with reference sequences from NCBI from Annelida, Arthropoda, Chordata, Cnidaria, Nematoda, Mollusca, Echinodermata, Porifera, and Platyhelminthes, revealed that PKA and PKC homologs are abundant throughout the Metazoa. Distinct isoforms of PKA catalytic subunits (Cα and Cβ), regulatory subunits (RIα and β; RIIα and β), and PKC (α, β, γ, δ, ε, η, θ, ι, and ζ) were clearly resolved within chordate clades. However, non-chordate sequences could not be classified into these specific isoforms.

### 2.1. PKA Catalytic Subunit

**Figure 1:**
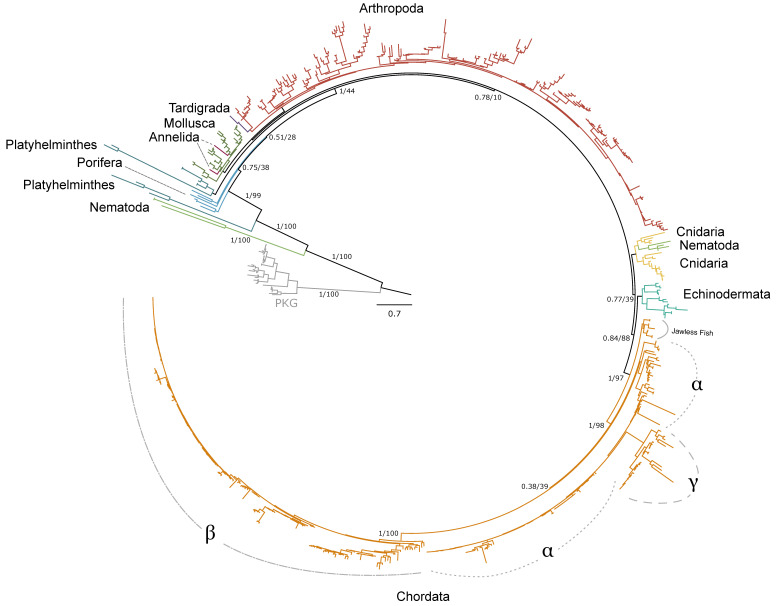
Phylogenetic relationships of the catalytic subunits of cAMP-dependent protein kinase among Metazoans. Tree is rooted by the PKG outgroup and was inferred using the JTT+I+G4 substitution model as identified by ModelFinder [41]. Branch support for outer branches is indicated by UltraFast bootstrap support as well as an approximate Bayes test (UFboot/aBayes). Within Chordata, the catalytic subunit isoform is denoted for α, β, and γ, as well as PKA-C homologs identified in jawless fishes, as characterized by Søberg and colleagues [40]. Line color by phyla is as follows: Porifera, light blue; Cnidaria, yellow; Platyhelminthes, dark blue; Mollusca, dark green; Annelida, maroon; Nematoda, light green; Arthropoda, red; Tardigrada, purple; Echinodermata, teal; Chordata, orange.

**Figure 2:**
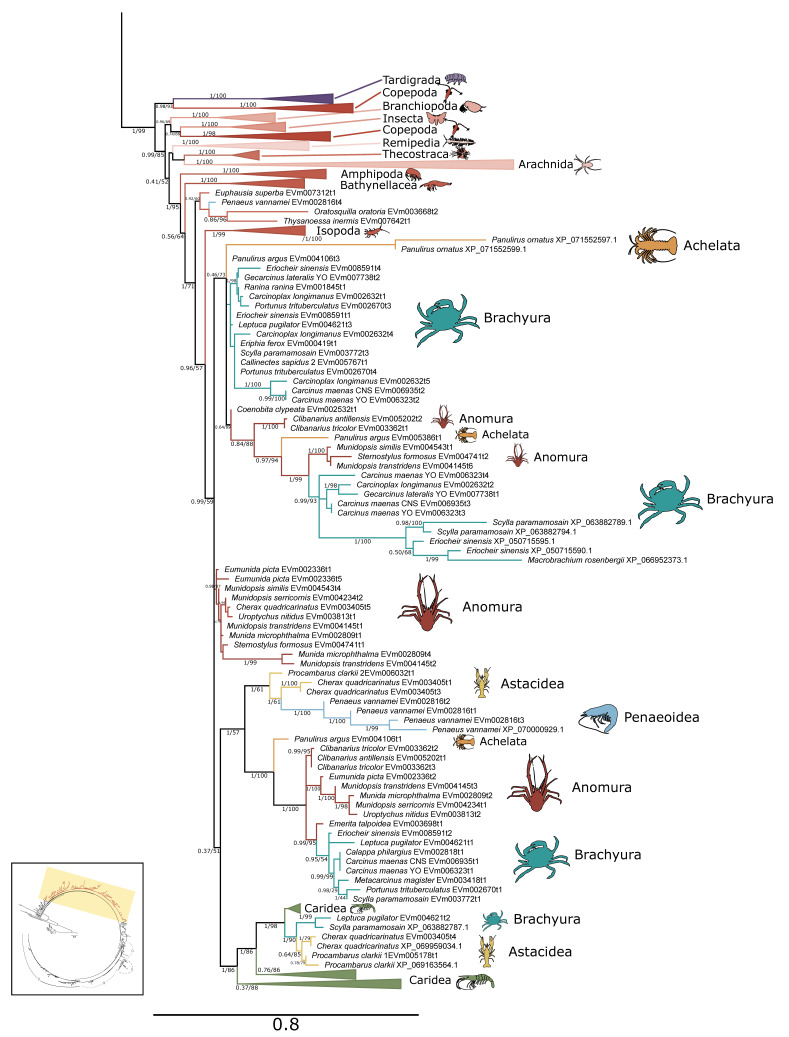
Phylogenetic relationship of PKA catalytic sequences among Arthropoda. Tree is an expanded view of the complete phylogeny presented in Figure 1, as indicated by the region highlighted in yellow in the inset. For visual clarity, orders other than Decapoda are collapsed, as well as caridean shrimp. Branch support is indicated for UFboot/aBayes. Taxa icons are from Phylopic.org; see Supplementary Files for complete references.

Within arthropods, 227 sequences from 150 species were identified as PKA-C; of these, 131 sequences were identified in decapod species (Table 1). Phylogenetic analysis of PKA-C across the Metazoa revealed distinct chordate isoforms (α, β, and γ), consistent with established classifications (Figure 1) [39,40]. By contrast, arthropod sequences did not segregate into these isoform-specific clades but instead formed several lineage-specific groups (Figure 1 and Figure 2).

**Figure 3:**
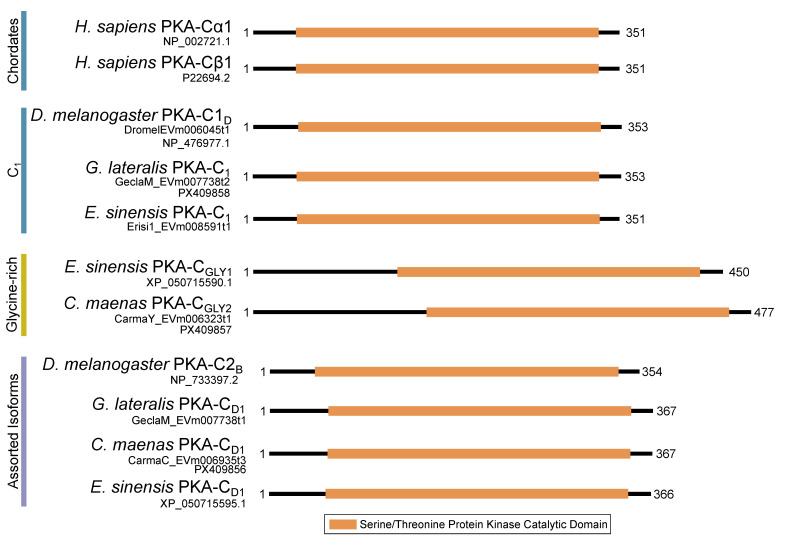
Domain organization of PKA catalytic subunits. To-scale schematic representing the length and position of the kinase domain in the catalytic subunit of PKA in humans, fruit flies, and true crabs (*G. lateralis*, *C. maenas*, and *E. sinensis*) when full-length sequences were identified.

**Figure 4:**
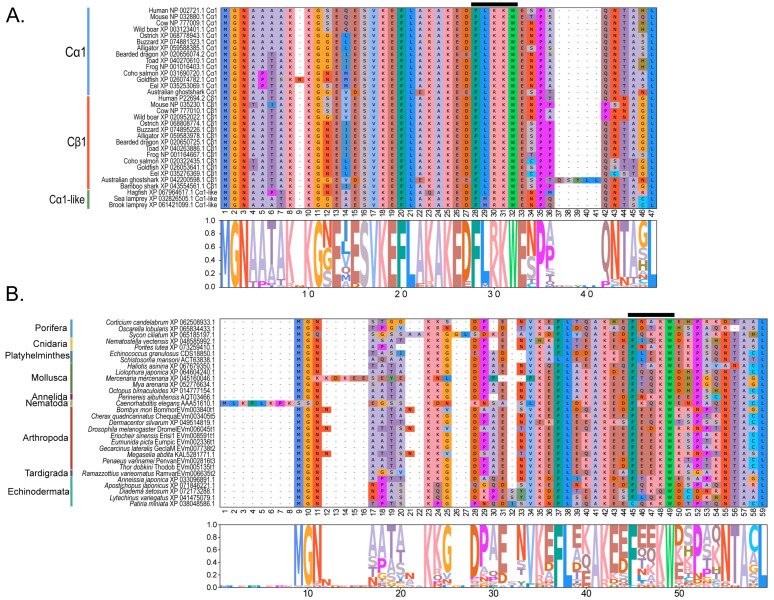
Multiple sequence alignments of the N-terminal region of PKA catalytic subunits among Metazoans. MSA color scheme corresponds to similarities in amino acid physiochemical properties. Full sequences were aligned using MAFFT-DASH. MSAs represent the complete sequence upstream of the kinase domain, as identified by NCBI-CDS. (**A**) Select chordate sequences representing PKACα and PKACβ, as well as the PKAC homologs identified in jawless fishes, identified by Søberg and colleagues [40]. (**B**) Select PKAC sequences from each major phylum included in this study that appear to be homologous to Cα and Cβ. The FxxxW motif is indicated with a black bar above MSAs. Consensus sequence is indicated below the alignment.

**Figure 5:**
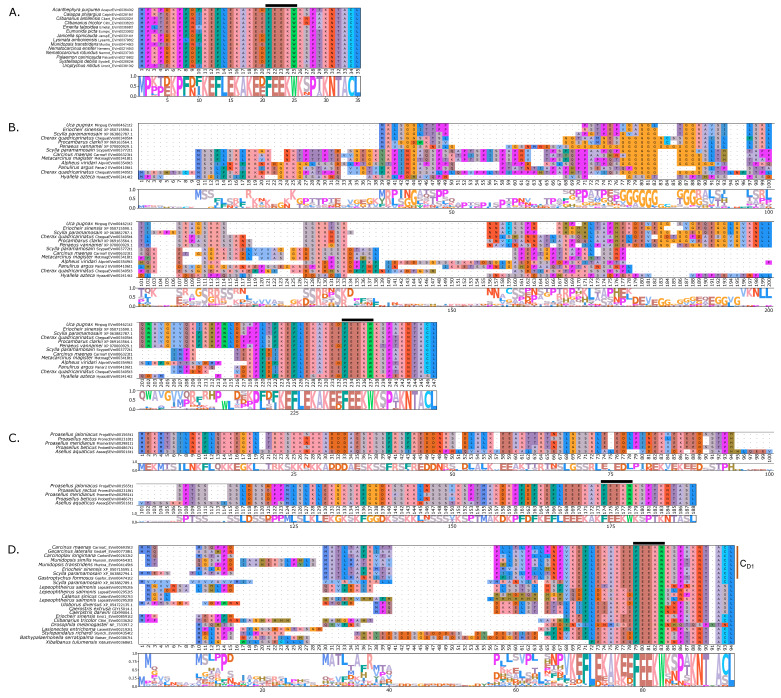
Multiple sequence alignments of the N-terminal region of PKA catalytic subunit among Arthropods. MSA color scheme corresponds to similarities in amino acid physiochemical properties. PKA catalytic subunits identified in arthropods fall into four major groups: (**A**) those only identified in decapods, (**B**) those containing long stretches of glycine residues, (**C**) those only identified in isopods, and (**D**) arthropod-specific sequences with no apparent similarities to groups (**A**–**C**). Within the arthropod-specific group, several decapod sequences shared a conserved motif MATL (reference positions #30 to #33) and are designated PKA-C_D1_. The FxxxW motif is indicated with a black bar above MSAs. Consensus sequence is indicated below the alignment.

Within the Brachyura, domain organization analyses identified multiple PKA-C isoforms consistent with the domain organization of model organisms, including humans and fruit flies (Figure 3). The sequences that were most similar at the larger-scale domain organization level (Figure 3; C_1_) also shared the most homology with chordate sequences in the N-terminal sequence upstream of the kinase domain (Figure 4). All major invertebrate phyla possessed PKA-C isoforms that were highly homologous to those characterized in chordates; these were designated PKA-C_1_, in accordance with established nomenclature in *Drosophila* (Figure 3 and Figure 4). The N-terminal consensus sequence, where x represents any amino acid, in chordates for all PKA-C isoforms was MGNxxxx[K/R] (Figure 4A). The N-terminal consensus sequence for PKA-C_1_ in invertebrates was MGNxxxxK, with a few species-specific exceptions (Figure 4B). Multiple sequence alignments (MSAs) of the N-terminal regions also indicated strong conservation of an FxxxW motif in PKA-C among metazoans (Figure 4 and Figure 5).

MSAs of the N-terminal region distinguished four major types of arthropod PKA catalytic subunits in addition to PKA-C_1_: (1) a decapod-specific type; (2) a glycine-rich type (PKA-C_GLY(1/2)_); (3) an isopod-specific type; and (4) an arthropod-specific type lacking clear similarity to other groups (Figure 5A–D, respectively). Within the arthropod-specific group, several decapod sequences have a conserved motif MATL[M/T/A]A[F/T] (Figure 5D, reference positions #30 to #36). Sequences sharing that motif were designated PKA-C_D1_ to indicate the first isoform to be characterized in the decapod clade outside of the ancestral-like PKA-C_1_ and the glycine-rich variant. Isoform variants, including PKA-C_GLY(1/2)_ and PKA-C_D1_, displayed unique domain organization due to the differences in lengths of the N- and C-terminal regions (Figure 3).

*Gecarcinus lateralis* and *Eriocheir sinensis* PKA-C_1_ subunits shared 86.9% and 87.2% identity and 91.2% and 91.5% similarity with human PKA-Cα, respectively (Table 2). Compared to human PKA-Cβ subunits, *G. lateralis* and *E. sinensis* PKA-C_1_ shared 86% and 86.3% identity and 90.9% and 91.2% similarity, respectively. PKA-C_GLY_ sequences in *Carcinus maenas* and the Pacific whiteleg shrimp *Penaeus* (*Litopenaeus*) *vannamei* were the most divergent among human, *Drosophila melanogaster*, and other decapod isoforms. *C. maenas* PKA-C_GLY2_ shared 63.2% identity and 65.6% similarity with *P. vannamei* PKA-C_GLY1_. *C. maenas* PKA-C_D1_ shared 81.8% identity and 87% similarity with *P. vannamei* PKA-C_1_ (Table 2).

### 2.2. PKA Regulatory Subunit

**Figure 6:**
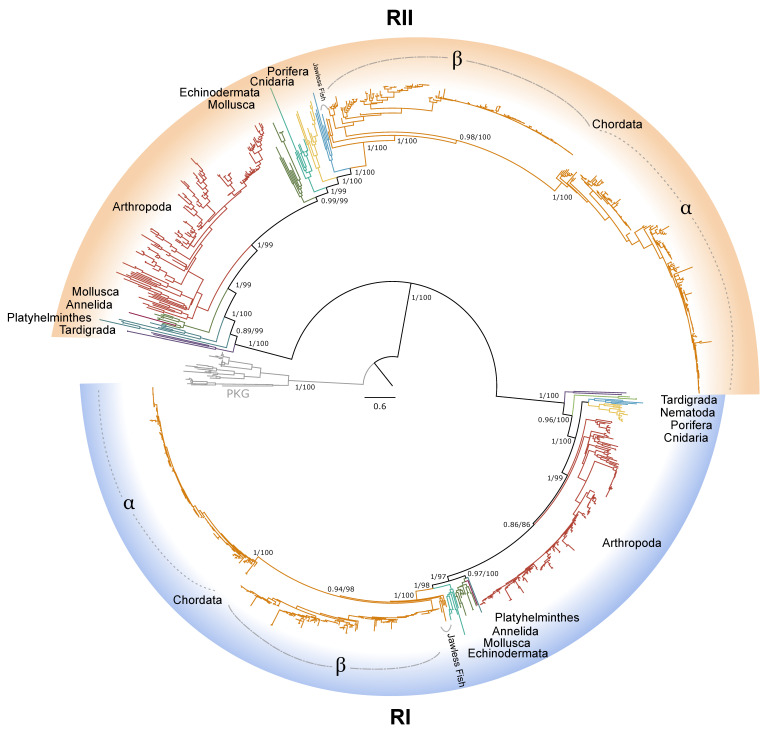
Phylogenetic relationships of the regulatory subunits of cAMP-dependent protein kinase among Metazoans. Maximum likelihood phylogenetic tree (JTT+I+G4) is rooted by cGMP-dependent protein kinase (PKG) outgroup (gray). PKA-RI (highlighted in blue) and PKA-RII (highlighted in orange) subunits form distinct clades. Within Chordata, the isoform of each subunit type is indicated, as well as PKA-RI and PKA-RII homologs identified in jawless fishes. Branch support for outer branches is indicated by UltraFast bootstrap support as well as an approximate Bayes test (UFboot/aBayes). Branch color indicates phyla as follows: Annelida, pink; Arthropoda, red; Chordata, orange; Cnidaria, yellow; Nematoda, light green; Mollusca, dark green; Echinodermata, teal; Porifera, light blue; Platyhelminthes, dark blue; Tardigrada, purple.

**Figure 7:**
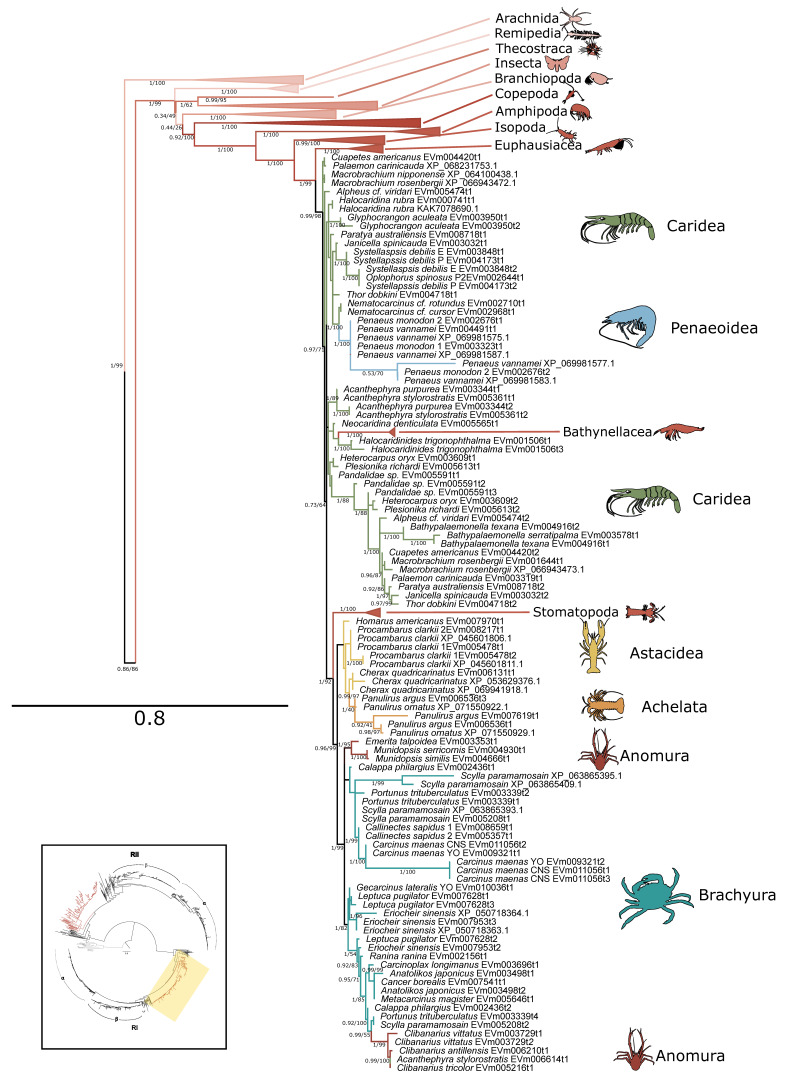
Phylogenetic relationship of PKA-RI sequences among Arthropoda. Tree is an expanded view of the complete phylogeny presented in Figure 6, as indicated by the region highlighted in yellow in the inset. For visual clarity, orders other than Decapoda are collapsed. Branch support is indicated for UFboot/aBayes. Taxa icons are from Phylopic.org; see Supplementary Files for complete references.

**Figure 8:**
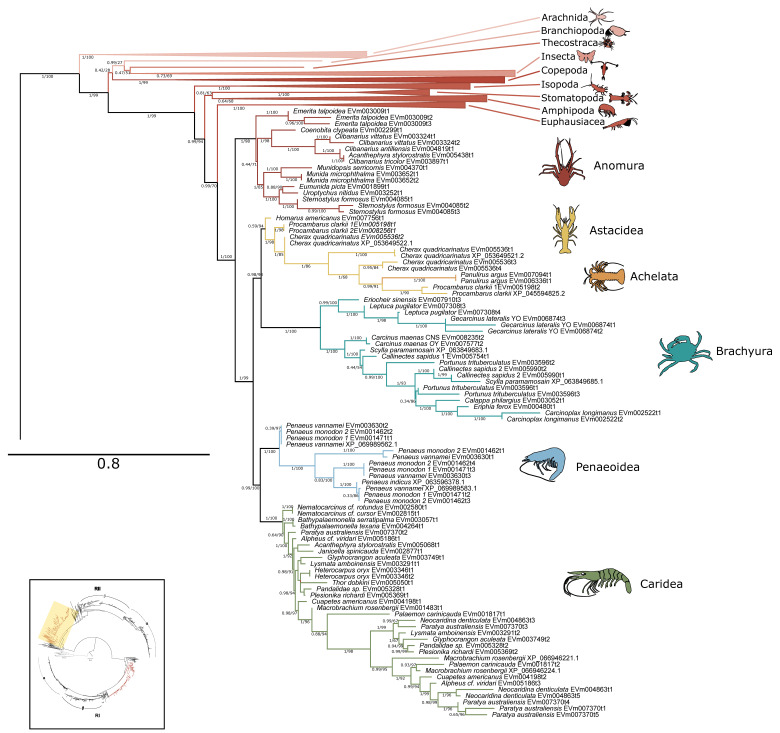
Phylogenetic relationship of PKA-RII sequences among Arthropoda. Tree is an expanded view of the complete phylogeny presented in Figure 6, as indicated by the region highlighted in yellow in the inset. For visual clarity, orders other than Decapoda are collapsed. Branch support is indicated for UFboot/aBayes. Taxa icons are from Phylopic.org; see Supplementary Files for complete references.

Within Arthropoda, 339 sequences from 150 species were identified as PKA regulatory subunits (Table 1). Of those, 105 were type I regulatory (RI) subunits and 97 were type II regulatory (RII) subunits in decapods (Table 1). Phylogenetic reconstruction of regulatory subunits resolved two well-supported clades corresponding to PKA-RI and -RII (Figure 6). In chordates, isoform-specific diversification within PKA-RI and -RII was apparent, whereas arthropod sequences generally grouped into broader, lineage-specific clusters (Figure 6, Figure 7 and Figure 8). PKA-RI sequences were identified in all major phyla included in this analysis, and PKA-RII sequences were identified in all phyla except Nematoda. Expanded views of PKA-RI and -RII sequences within the Arthropoda showed that most decapod species expressed multiple isoforms of each type of regulatory subunit. PKA-RI subunits formed monophyletic clades for all Malacostracan orders and infraorders, except the Caridea and Anomura (Figure 7). PKA-RII subunits formed monophyletic clades for all classes within the Arthropoda (Figure 8).

**Figure 9:**
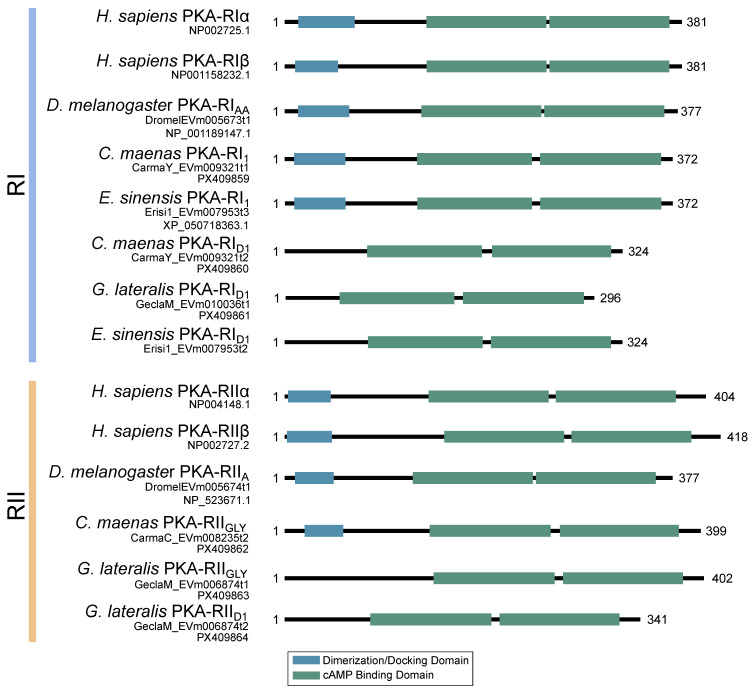
Domain organization of the regulatory subunits of PKA. To-scale schematic representing the length and position of the dimerization/docking domain (blue) and the two cAMP-binding domains (green) in the regulatory subunit of PKA in humans, fruit flies, and true crabs (*G. lateralis*, *C. maenas*, and *E. sinensis*) when full-length sequences were identified. Multiple regulatory subunit isoforms were identified in brachyurans, including complete RI and RII sequences, as well as isoforms that did not contain a dimerization domain identifiable by NCBI-CDS.

**Figure 10:**
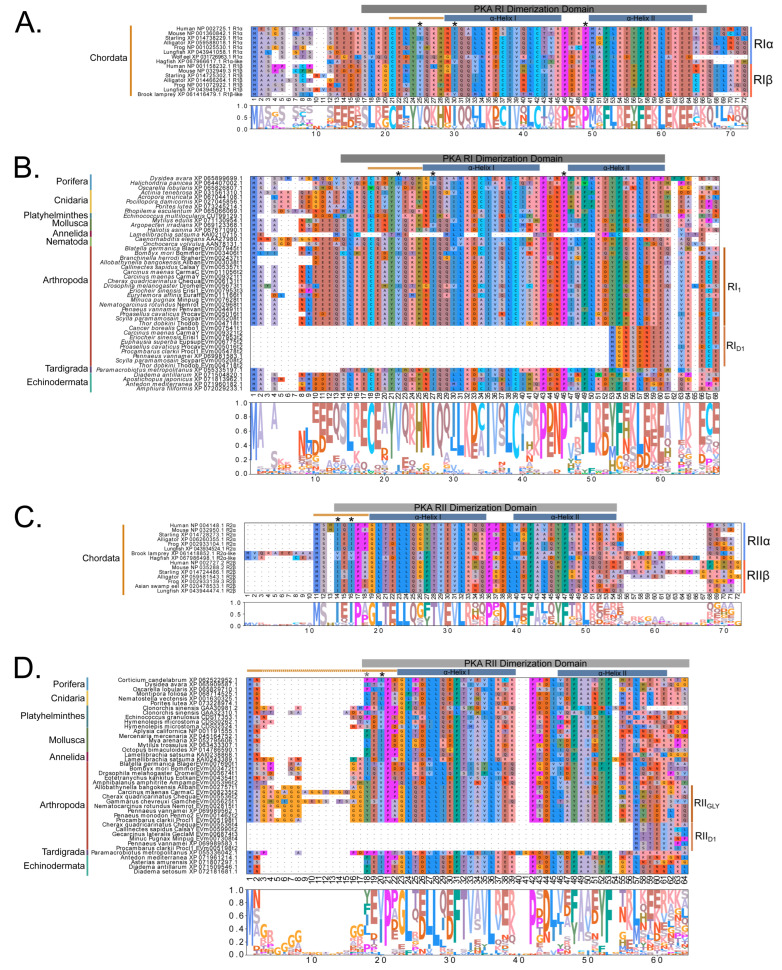
Multiple sequence alignments of the dimerization domain of PKA regulatory subunits in Metazoans. MSA color scheme corresponds to similarities in amino acid physiochemical properties. Full sequences were aligned using MAFFT-DASH; dimerization domains were identified by NCBI-CDS and are indicated with gray bars for each group. A-helices are indicated by blue bars, the motif that distinguishes RI and RII D/D domains (horizontal orange line), and residues that are essential for AKAP binding (asterisk *) were identified based on homology with D/D domains as previously annotated (Peng et al., 2015 [14]; Dahlin et al., 2021 [42]). Select PKA-RI sequences from chordates (**A**) and invertebrates (**B**) and select PKA-RII sequences from chordates (**C**) and invertebrates (**D**).

**Figure 11:**
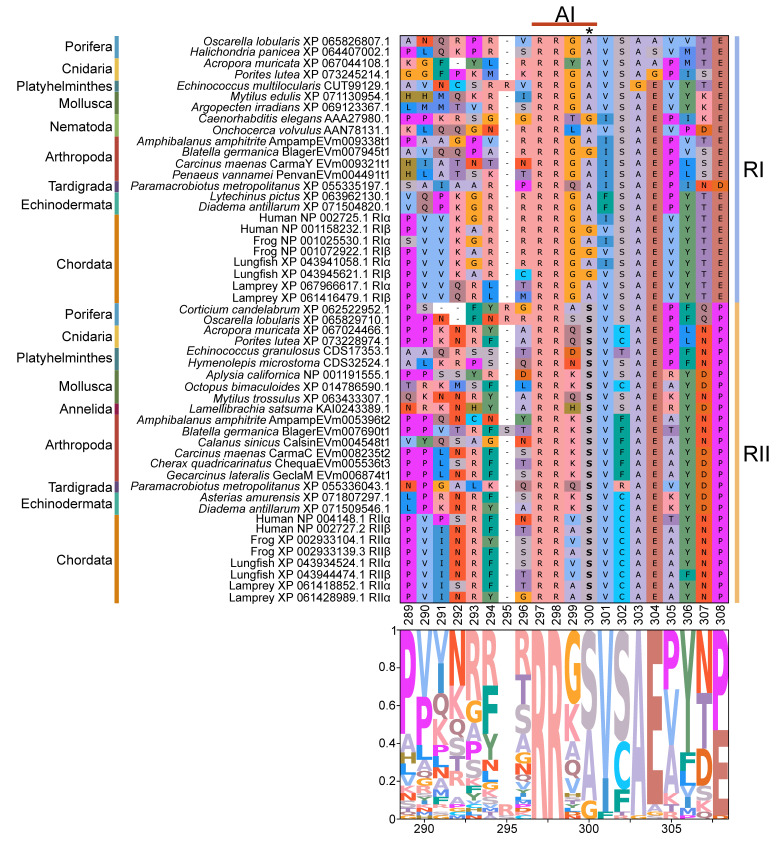
Multiple sequence alignment of the autoinhibitory substrate within PKA regulatory subunits among Metazoans. MSA color scheme corresponds to similarities in amino acid physiochemical properties. The autoinhibitory site (AA positions 297–300) is located between the D/D domain and the kinase domain in PKA regulatory subunits. Subunits are sorted by phyla, as indicated on the left, and type, as indicated on the right (RI, light blue; RII, yellow). RI and RII AI sites are distinguished at residue 300 (in this alignment, as indicated by an asterisk *). The RI motif is RRx[A/G] and the RII motif is RRxS, where x represents any amino acid.

Identification of conserved domains in brachyuran crabs confirmed the presence of multiple regulatory isoforms, some of which lacked an identifiable dimerization domain (D/D; Figure 9). Alignments of the D/D, or the full sequence N-terminal to the first cAMP-binding domain when a D/D was not identified, revealed that invertebrate PKA-RI and -RII subunits retained the overall structural motifs of chordates but also displayed notable sequence divergence from chordates (Figure 10). Within PKA-RI, a proline residue (Figure 10A, reference position #49) important for AKAP binding in chordates was conserved in all invertebrate sequences that contained an identifiable D/D domain (Figure 10B, reference position #46). In PKA-RII, the IxI or VxV motifs (Figure 10C, reference positions #14 to #16), which are important for AKAP binding in chordates, were not highly conserved in invertebrate sequences (Figure 10D, reference positions #18 and #20). A PKA-RII isoform with a glycine-rich N-terminal region was identified in arthropods and designated PKA-RII_GLY_. The N-terminal region of PKA-RII_GLY_ was longer than that of other phyla, and glycine was particularly enriched among decapods relative to other arthropod species, which was similar to PKA-C_GLY_. The autoinhibitory (AI) site was completely conserved among metazoans, with the RRx[G/A] motif for PKA-RI and RRxS motif for PKA-RII (Figure 11, reference positions #297 to #300).

The *C. maenas* PKA-RI_1_ subunit shared 67.5% and 69% identity and 79.8% and 80.3% similarity with human PKA-RIα and -RIβ, respectively (Table 3). The *C. maenas* PKA-RII_GLY_ subunit shared 48.5% and 46.7% identity and 63.2% and 61.8% similarity with human PKA-RIα and -RIβ, respectively (Table 4). When compared to *Drosophila*, decapod PKA-RII subunits shared about 53 to 59% identity and 66 to 73% similarity. A full-length PKA-RII subunit of any isoform was not identified in *E. sinensis*, which was likely a result of transcript assembly methods rather than the absence of PKA-RII in that species.

### 2.3. PKC Sequences

**Figure 12:**
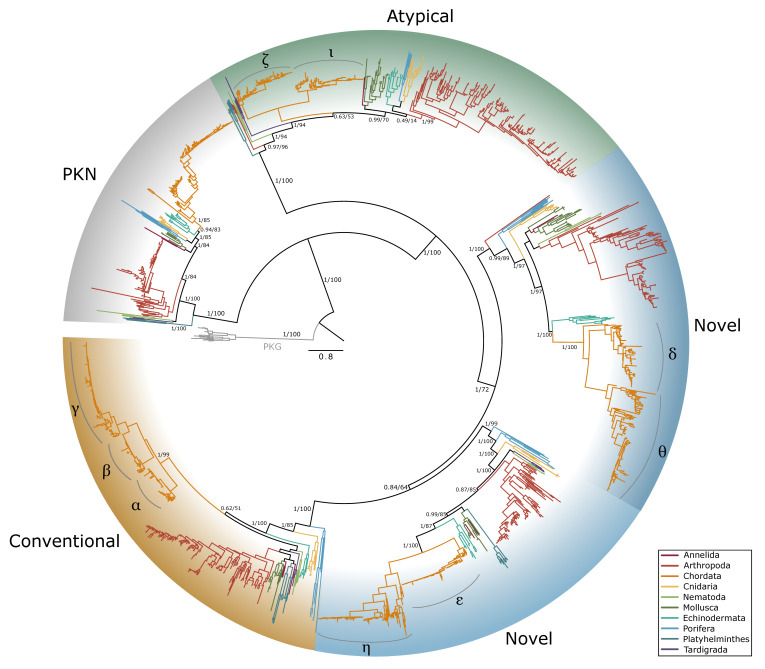
Phylogenetic relationship of protein kinase C among Metazoans. Maximum likelihood phylogenetic tree (JTT+I+G4) is rooted by PKG as an outgroup. PKC-related proteins PKD (light gray shading) and PKN (dark gray shading) were identified as distinct from the conventional (gold shading), novel (blue shading), and atypical (green shading) PKCs. Within Chordata, isozymes within each PKC subfamily are indicated. Branch support for outer branches is indicated by UltraFast bootstrap support as well as an approximate Bayes test (UFboot/aBayes). Branch color indicates phyla as follows: Annelida, pink; Arthropoda, red; Chordata, orange; Cnidaria, yellow; Nematoda, light green; Mollusca, dark green; Echinodermata, teal; Porifera, light blue; Platyhelminthes, dark blue; Tardigrada, purple.

A total of 678 PKC and PKN sequences were identified in 173 species of arthropods, including 153 conventional, 106 novel delta-like, 100 novel epsilon-like, 204 atypical PKCs, and 115 PKNs (Table 5). The phylogenetic tree of PKC sequences across Metazoa recovered the five canonical PKC subfamilies: conventional (cPKC), novel (nPKC), delta-like, nPKC epsilon-like, atypical (aPKC), and PKN (Figure 12). Within decapods, 315 total sequences were identified in 52 species, including 62 cPKC, 34 nPKC delta-like, 44 nPKC epsilon-like, 98 aPKC, and 77 PKNs (Table 5). Within chordates, isozymes within each subfamily were clearly resolved, while arthropod PKCs clustered into lineage-specific groups, indicating independent diversification. Homologs of each PKC subfamily and PKN were identified in each phyla examined, except for nPKC delta-like, which was not identified in Platyhelminthes and Tardigrada (Table 5).

**Figure 13:**
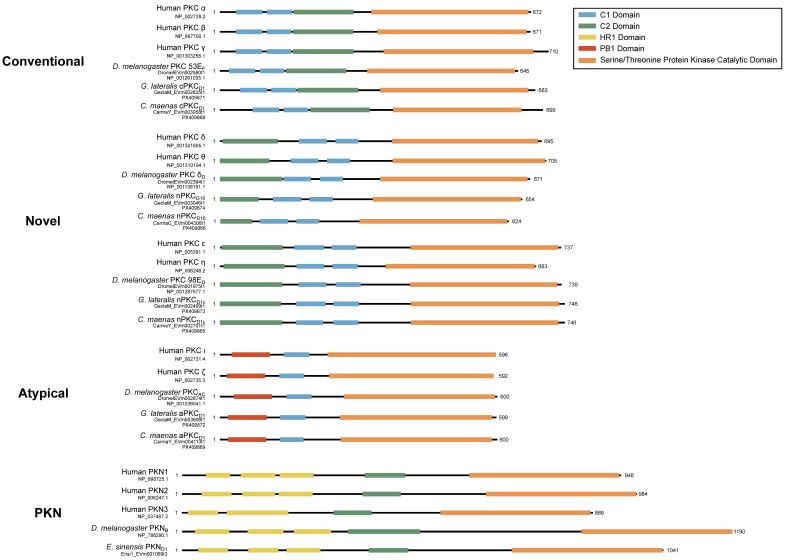
Domain organization of PKC and PKN sequences. To-scale schematic representing the length and position of the C1 (blue), C2 (green), HR1 (yellow), PB1 (red), and kinase (orange) domains in the fruit flies and true crabs (*G. lateralis*, *C. maenas*, and *E. sinensis*) when full-length sequences were identified. Arthropod PKC sequences follow the conventional domain structure for each subfamily characterized in model organisms.

**Figure 14:**
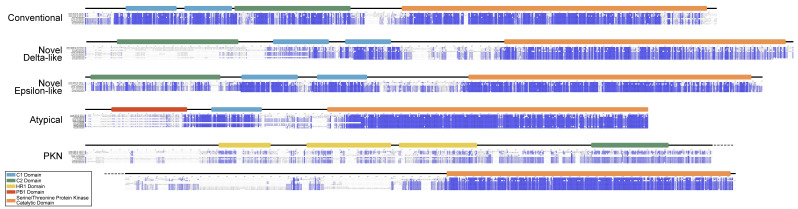
Percent identity of PKC multiple sequence alignments. Multiple sequence alignments of the PKC sequences in Table 6. Sequences represent human, *D. melanogaster*, *C. maenas*, *G. lateralis*, *E. sinensis*, *C. quadricarinatus*, and *P. vannamei* from top to bottom. Shading indicates percent identity, with dark purple representing > 80% similarity, medium purple > 60% similarity, light purple > 40% similarity, and white < 40% similarity between aligned sequences in each PKC subfamily. Positions of conserved domains are aligned with the human sequences for PKCα, PKCδ, PKCε, PKCι, and PKN1, respectively.

Domain organization analyses of brachyuran PKC subunits revealed canonical structures consistent with each subfamily described in model organisms, including human and *Drosophila* (Figure 13). MSAs revealed that decapod PKCs, while similar in overall domain organization, could not be sorted into a homologous chordate-defined isozyme within each subfamily (Figure 14). For brevity, percent identity to human PKC homologs was only calculated for *D. melanogaster*, *C. maenas*, *G. lateralis*, *E. sinensis*, *Cherax quadricarinatus*, and *P. vannamei* (Table 6). At least one sequence homologous to chordate cPKCs was identified in each decapod species. However, decapod cPKC sequences did not share homology within the regions/residues used to distinguish cPKCα and β isozymes among chordates, and the percentage of residue identity did not differ greatly when comparing to cPKCα or cPKCβ (Table 6). Decapod sequences were nominally more similar to human nPKCε and aPKCι than to nPKCη and aPKCζ, respectively (Table 6).

While decapod sequences cannot be neatly defined within the PKC naming conventions established for chordates, there is strong protein sequence conservation of the kinase domain within each subfamily (Figure 14). cPKCs also showed strong (>80%) conservation of the C1 and C2 domains between humans, *Drosophila*, and decapods. nPKC delta-like were poorly conserved within the C2 domain and moderately (>60%) conserved within the C1 domains. nPKC epsilon-like showed strong conservation among the C1 domains and moderate conservation of the C2 domain. aPKCs had a strongly conserved C1 domain, while the PB1 domain was poorly conserved (<40%). PKNs had a moderately conserved C2 domain, while the three HR1 domains were relatively poorly conserved.

## 3. Discussion

Current naming conventions used to distinguish subfamilies and isoforms of PKA and PKC are based on genetic and biochemical studies conducted primarily on chordates, particularly human, mouse, and bovine [5,43,44,45]. Later studies expanded this characterization to include invertebrate lineages, particularly *Drosophila* and *Aplysia* [43,46,47,48,49,50,51,52,53]. Analyses of the evolution and divergence of AGC kinases have revealed that large sequence variation exists between chordate and non-chordate taxa, such as the Pacific whitelegged shrimp, as reviewed in [2]. While phylogenetic analyses of PKA and PKC have expanded to include non-chordate lineages, they are often limited by the number of available non-chordate sequences [17,39,40,52], use a limited number of characterized (typically chordate) reference sequences [54,55], or apply chordate naming conventions to paralogs instead of orthologs [54,56]. Application of the Eukaryotic Genome Annotation Pipeline [57] has vastly expanded the availability of invertebrate sequences in the NCBI RefSeq database. However, because the previously annotated sequences available for reference are primarily those of model species, misidentification at the isoform level by the annotation pipeline can lead to downstream errors if not validated by users.

The current study established an extensive phylogenetic analysis of metazoan PKA (Figure 1 and Figure 6) and PKC (Figure 12) protein sequences. A previous comparative analysis established that arthropods and chordates retain two PKG genes [31]. Together, these analyses provide a blueprint for characterizing AGC kinase sequences in non-chordate lineages. Our results show that while the core structures of PKA and PKC are conserved across the Metazoa, arthropod sequences exhibit significant divergence from chordate isoforms. This divergence underscores the importance of characterizing kinases in non-model taxa to better understand the evolutionary history and functional diversity of these signaling enzymes.

Chordate PKA catalytic subunits form distinct Cα, Cβ, and Cγ isoforms, whereas non-chordate lineages do not fall into these isoform-specific clades. However, MSAs indicate that invertebrate lineages retain a PKA-C subunit with significant homology to PKA-Cα1 and PKA-Cβ1 identified in chordates (Figure 4). In order to establish a consistent nomenclature across phyla, we propose that these invertebrate catalytic subunits, as identified by the N-terminal consensus sequence MGNxxxxK, be designated PKA-C_1_ (Figure 4B). This annotation is consistent with the established convention used for the same catalytic subunit isoform (DC1) in *Drosophila* [51].

Phylogenetic analysis of PKA-C sequences reflects the evolutionary history of the Metazoa. The sequence identity of the N-terminal region, which extends from the N-terminus to the beginning of the kinase domain, among PKA-C_1_, -Cα1, and -Cβ1 is striking. PKA-C genes underwent an ancient duplication in an early eukaryotic ancestor of metazoans and fungi, with one gene representing the canonical PKA-C subunits and the other representing a closely related protein called PRKX [14]. Subsequent parallel duplications in distinct lineages resulted in specialized isoforms [14]. The genes encoding PKA-Cα and -Cβ in vertebrates arose from a duplication of an ancestral catalytic subunit gene between the divergence of Agnatha (jawless fishes) and Chondrichthyes (cartilaginous fishes) [4,39,40]. Taken together, the data suggest that invertebrate PKA-C_1_ and chordate PKA-Cα and -Cβ share an ancestral gene retained across the Metazoa (Figure 1).

Arthropod-specific diversification of PKA-C occurred in the N-terminal region. Decapods showed lineage-specific expansion, including glycine-rich variants, designated PKA-C_GLY1_ and -C_GLY2_, which contain an elongated, glycine-rich N-terminal. Within decapods, PKA-C_D1_ shows further species-specific divergence primarily in the sequence upstream of the MATL motif (Figure 5D). In chordate PKA-Cs, myristoylation of glycine in position #2 of the N-terminus (G2) targets the activated subunit to the membrane after dissociation from the regulatory subunit [58]. The G2 was conserved in PKA-C_1_ in species from all phyla examined except Nematoda (Figure 4B). By contrast, the G2 was only identified in four sequences from two species of arthropod-specific PKA-Cs that do not fall into either the PKA-C_GLY_ or PKA-C_D1_ group (Figure 5D; see *Lepeophtheirus salmonis* and *Bathypalaemonella serratipalma*). N-terminal diversification of PKA-C within decapods may alter the regulation, localization, or protein–protein interactions relative to those characterized in chordate species. PKA-C_1_, PKA-C_GLY(1/2)_, and PKA-C_D1_ sequences only differed in the N-terminal region (Figure 5), whereas the protein sequence from the beginning of the catalytic domain to the C-terminus was 100% identical among different isoforms identified within the same decapod species (see Supplementary Data for MSAs). Moreover, the FxxxW motif in the N-terminal region was entirely conserved in all arthropod-specific catalytic subunit sequences (Figure 4 and Figure 5). This motif acts as a hydrophobic anchor that binds to a pocket within the kinase core, stabilizing the catalytic domain in its active conformation [4,59]. Conservation of this motif in the otherwise divergent N-terminal region indicates that the core kinase function in decapod PKA-Cs is conserved. Further examination of genomic data can clarify whether these decapod isoforms arise from alternative splicing of a single gene, as the conservation in protein sequence suggests.

Two major clades of PKA regulatory subunits corresponding to type RI and RII subunits were resolved. The well-supported split between PKA-RI and -RII in the phylogenetic analysis (Figure 6) supports the divergence of the two regulatory subunits as arising before the last metazoan common ancestor [9,42,60]. As observed with PKA-C, PKA-R could not be further resolved into chordate α or β types within PKA-RI and -RII clades for non-chordate lineages (Figure 6). Also consistent with PKA-C, non-chordate PKA-RI and -RII were distinguished by the N-terminal sequence upstream of the first cAMP-binding domain. Within the N-terminal region, the D/D domain is the site of dimerization of PKA-R subunits and also determines the specificity of PKA-RI and -RII binding to A-kinase anchoring proteins (AKAPs), which regulate spatiotemporal localization and three-dimensional configuration of PKA-AKAP complexes [9,10,13,14,15,42,61,62]. AKAPs bring the PKA holoenzyme within proximity of both its substrates as well as its ligand in a mechanism that compartmentalizes cAMP signaling within a cell [9,10,13,14,15]. Recent analysis of AKAP evolution determined that early metazoans express relatively few AKAPs compared to chordate lineages since the appearance of vertebrates [9]. Early evolving PKA-AKAP complexes laid the foundation for the highly regulated cellular compartmentalization of cAMP signaling observed in higher-order metazoans [9,14,42,63]. To our knowledge, AKAPs have not been characterized in arthropods. Characterization of the D/D domain in PKA-R subunits is needed in order to identify corresponding AKAPs. The data presented here provides the basis for further study on evolutionary relationships between invertebrate AKAPs and PKAs.

The expansion of AKAP sequences early in vertebrate evolution is presumed to be associated with the corresponding increase in cellular and organismal complexity. However, non-chordate clades have their own set of circumstances that require unique cellular adaptations, including but not limited to sessile life stages, metamorphosis and molting, and thermal stress. Among decapods, D/D domains with a relatively high degree of homology to those characterized in chordates were identified. Notably, one PKA-RII AKAP-binding residue important in chordates (reference positions #14 and #18 in Figure 10C,D, respectively) was poorly conserved among invertebrate species. Within arthropods, the N-terminal region of PKA-RII_GLY_ was longer than that of other phyla, and glycine was particularly enriched among decapods relative to other arthropod species, as observed in PKA-C_GLY(1/2)_. Moreover, truncated PKA-RI and -RII sequences that lacked a D/D domain were expressed in decapods. Whether truncated subunits lacking a D/D domain are capable of dimerizing or interacting with AKAPs should be explored. Given the breadth of divergence apparent in PKA-R subunits across the Metazoa, it is likely that unique AKAPs may interact with lineage-specific isoforms of these PKA-R subunits.

PKA-R contains an autoinhibitory (AI) site in the linker region between the D/D and the first cAMP-binding domains [11,12]. The AI site interacts with PKA-C by filling the active site cleft in the kinase domain. In PKA-RI, the AI site is a pseudosubstrate consisting of the RRx[A/G] motif. In PKA-RII, the AI site is an autophosphorylation substrate consisting of the RRxS motif, where the serine residue is phosphorylated by PKA-C in the heterotetrameric PKA holoenzyme [11,15,16,52,64,65]. These AI motifs were entirely conserved among metazoan PKA-RI and -RII subunits (Figure 11). Taken together, these results suggest that the overall dimerization, AKAP-binding, cAMP-binding, and autoinhibitory framework of PKA-R subunits are conserved among metazoans, but that lineage-specific diversification in non-chordates may alter their functional properties. Such divergence could modify how regulatory and catalytic subunits interact, alter their sensitivity to cAMP concentrations, determine the magnitude of the cellular response to cAMP, and/or affect subcellular localization. Biochemical studies of decapod PKA-C and PKA-R should be conducted to understand the functional consequences of these novel N-terminal sequences.

Five distinct PKC protein clades were identified by phylogenetic analyses (Figure 12). These clades corresponded to the five subfamilies found in chordates: PKN, conventional (cPKC), atypical (aPKC), and two novel (nPKC) subfamilies, delta-like and epsilon-like. The PKC tree was rooted from PKG, which diverged earlier than PKCs and shares only the kinase domain, which is highly conserved across AGC kinases [2,17,37]. This phylogeny supports the assertion that PKN is basal to the other PKC subfamilies [17]. With the exception of nPKC delta-like in Platyhelminthes and Tardigrada, at least one sequence from each of the major metazoan phyla was identified in each subfamily. This result is consistent with a previous analysis that included one species each from sponge (Porifera), roundworm (Nematoda), fruit fly (Arthropoda), and sea urchin (Echinodermata), and was rooted from a single yeast PKC1 sequence [17]. The absence of nPKC delta-like sequences in Platyhelminthes and Tardigrada may represent a gene loss but also may be a result of the relatively limited sequence data available for those phyla.

In basal metazoan lineages, PKCs within each subfamily cannot be assigned as orthologous to the isoform classification established in chordates. In order to distinguish decapod sequences from those in chordates, we propose a naming convention that uses the subfamily letter (conventional, c; novel, n; atypical, a) and is followed by the letter D for Decapoda. The Greek symbol δ or ε follows the letter D to distinguish novel delta-like or epsilon-like subfamilies, respectively. Sequences within each isoform are numbered in the order in which they are characterized (Table 7). For example, the conventional decapod PKC is designated cPKC_D1_; the novel PKCs are designated nPKC_D1δ_ and PKC_D1ε_; the atypical PKC is designated aPKC_D1_; and PKN isoforms are designated PKN_D1-3_ (Table 6 and Table 7). While conservation of the C1, C2, PB1, and HR1 domains between human and decapod varied by isoform, the kinase domain was strongly conserved (Figure 14). Within each PKC subfamily, regions between conserved domains show the greatest divergence. These data suggest that, while regulation may differ between decapods and chordates, kinase function is retained. Although this classification does not reveal the conservation or identity of ancestral PKC genes, it can support future work to determine the functional characteristics of decapod PKC isoforms.

## 4. Materials and Methods

### 4.1. Phylogenetic Analysis

Phylogenetic analysis of both PKA and PKC began by collecting a broad range of chordate protein reference sequences classified as *Homo sapiens* orthologs by NCBI for each PKC isozyme (α, β, δ, γ, ε, η, ι, ζ, and PKN) or PKA subunit (Cα1, Cβ1, RIα, RIβ, RIIα, and RIIβ). To broaden the phylogenetic depth of our analysis, individual NCBI protein BLAST (BLASTp) searches of human and mouse sequences were run with taxonomy exclusive to Porifera, Cnidaria, Platyhelminthes, Mollusca, Annelida, Nematoda, Arthropoda, Tardigrada, and Echinodermata [66]. Protein reference sequences selected from NCBI BLASTp results were only included for sequences with E-values ≤ 1 × 10^−70^, percent identity above 60%, and at least one conserved domain identified in the Conserved Domain Database (CDD) [67].

Protein reference sequences were concatenated for PKA-C, PKA-R, or PKC subfamily and used as a query for local BLAST (version 2.12.0) [66] searches against the CrusTome database (version 0.1.0) [38] to identify orthologous sequences with broad phylogenetic representation among pancrustaceans, as previously described [31,38,68]. CrusTome BLAST results with E-values ≤ 1 × 10^−120^ were concatenated with respective query reference sequences and aligned using MAFFT-DASH (version 7.508) [69] with 1000 maximum iterations and the flags ‘--originalseqonly’ to exclude added homologs used for alignment from the output, and ‘--genafpair’ to use the E-INS-i pairwise alignment algorithm. Multiple sequence alignments (MSAs) were trimmed using ClipKit (version 1.3.0) [70] with the smart-gap parameter to retain phylogenetically informative sites. IQ-TREE (version 1.6.12) [71] was used to create initial maximum likelihood phylogenetic trees of aligned and trimmed sequences with estimated evolutionary models using ModelFinder (-msub nuclear, all other settings left as default) [41] and 1000 ultrafast bootstrap approximations (UFboot) [72].

Initial datasets were refined by removing sequences arising from contamination and incomplete sequences. Such sequences were identified by long branch lengths or abnormal grouping (e.g., arthropod sequences within nematode clades) in initial trees and were examined with NCBI BLASTp and the CDD. The results of BLASTp searches were used to exclude sequences from nematodes, trematodes, or mites that frequently infect crustacean species ([73,74,75]; personal observations) by identifying contaminant species, rather than decapods, within the top hits. Sequences with fewer than two complete conserved domains were removed from further analysis for PKA-R and PKCs to eliminate the inclusion of other protein types with shared conserved domains (e.g., other cAMP-binding proteins or kinases).

Refined datasets of each PKC subfamily were concatenated into a single PKC FASTA file. Select PKG sequences from previous characterization [31] were included in each dataset as an outgroup. Refined datasets were aligned using MAFFT-DASH (--originalseqonly --genafpair --maxiterate 10000). Final phylogenies were inferred with IQ-TREE using the JTT+I+G4 substitution model based on consistent selection in initial trees, and branch support for phylogenetic relationships was estimated with UFboot approximation with 10,000 iterations [72] alongside an approximate Bayes (aBayes) test [76,77]. Final tree files, input (FASTA) files, alignments, and code used for phylogenetic analysis are available in the Supplemental Data.

### 4.2. Multiple Sequence Alignments

Sequences among species from each phylum represented were selected for representative MSAs of PKA-C, PKA-RI, and PKA-RII based on completeness. Sequences were aligned using MAFFT-DASH with 10,000 iterations as described for phylogenetic inference. MSAs were visualized using the *plot_msa.py* script [78] available at https://github.com/invertome/scripts/tree/main/plots (accessed on 8 October 2024). Percent identity of PKC MSAs was visualized in JalView (version 2.11.5.0) [79].

### 4.3. Identity and Similarity Calculations

Sequence pairs were aligned with MAFFT-DASH, as described above [69]. Percent identity and sequence similarity, based on groups of amino acids with shared properties, was calculated with the ‘Ident and Sim’ feature from the Sequence Manipulation Suite [80]. Amino acid groups for similarity calculations were as follows: GAVLI, FYW, CM, ST, KRH, DENQ, and P [80].

## 5. Conclusions

Phylogenetic and bioinformatic analyses of CrusTome and GenBank databases have yielded the most comprehensive catalog of invertebrate PKA and PKC protein sequences to date. Previous phylogenetic analyses have emphasized chordate PKAs [14,39,40,52] and PKCs [1,2,17,21,81]. The data presented herein represents a robust assemblage of protein sequences encoding both conserved (ancestral) and divergent PKA catalytic subunits, PKA regulatory subunits, and PKCs in Porifera, Cnidaria, Platyhelminthes, Mollusca, Annelida, Nematoda, Arthropoda, Tardigrada, and Echinodermata. This curated dataset provides a resource for invertebrate sequence characterization in taxa that are not well represented in the literature.

Although genomic resources for crustaceans are expanding, most brachyuran genomes remain at the scaffold level, limiting their utility for synteny or duplication analyses. By contrast, protein sequences represent the direct functional products of genes and provide the clearest insight into the structural features that mediate kinase activity. By focusing on conserved domains, motifs, and sequence variation at the protein level, this study establishes a strong foundation for inferring functional similarities and differences between decapods and well-characterized chordate kinases. As higher-quality genomic assemblies become available, future work can integrate gene-level analyses with the protein-based framework presented here to clarify the evolutionary trajectories of kinase subfamilies. Ultimately, our protein-centric approach ensures that functional interpretation remains directly tied to the signaling roles these enzymes have in decapod physiology.

This study shows that arthropod PKA and PKC sequences cannot simply be assigned to chordate isoform nomenclature. We propose a nomenclature for decapod crustacean PKA catalytic (PKA-C_1_, -C_GLY1_, -C_GLY2_, -C_D1_) and regulatory (PKA-RI1, -RI_D1_, -RII_GLY_, and RII_D1_) subunits and PKC types (cPKC_D1_, nPKC_D1δ_, nPKC_D1ε_, aPKC_D1_, and PKN_D1-3_) that can be used in classifying PKA and PKC sequences in other invertebrates (Table 7). Decapod PKA and PKC sequences reveal conservation of the catalytic machinery and domain organization, alongside lineage-specific diversification in motifs and domains associated with dimerization and spatiotemporal localization. Such lineage-specific variation likely reflects adaptation to arthropod-specific, and more directly, decapod-specific, life history traits such as those associated with molting, reproduction, stress responses, and osmoregulation [27,28]. These divergent sequences also highlight the importance of the functional characterization of proteins within decapods, as established mammalian annotations may not accurately reflect their functions in crustaceans.

Early studies examining the physiological roles of AGC kinases in decapod physiology did not include bioinformatic characterization of the protein sequences. Multiple isoforms of decapod PKA and PKC had not yet been identified, and these studies relied on the assumption of conserved function with mammalian (primarily human, mouse, rat, and bovine) homologs [32,56,82,83,84,85,86,87,88]. In the Brachyura, PKA, PKG, and PKC control YO ecdysteroid synthesis and secretion, and therefore molting. This expanded characterization of decapod-specific PKA and PKC isoforms, along with recent advances in understanding the compartmentalization of PKA [15] and PKC [89] signaling pathways, provides new insight into their roles in ecdysteroidogenesis. Given the diversification of PKA-C and PKA-R subunits identified in this study, it is possible that a crustacean-specific PKA isoform regulates ecdysteroid synthesis in the crustacean YO and the insect prothoracic gland [9,10,13,14,15,78]. Likewise, PKC subfamilies may play unique roles within the YO, as previous studies using Ca^2+^ and a phorbol ester (PMA) did not distinguish between conventional and novel PKCs [90]. PKN and atypical PKCs likely also play important roles, as these sequences are expressed in *G. lateralis* and *C. maenas* (CarmaY) YO transcriptomes (Table 7) [38,91,92,93]. Taken together, these data indicate that YO regulation is far more complex than was previously appreciated and provide a foundational resource for the functional characterization of PKA and PKC isoforms.

## Figures and Tables

**Table 1:** Taxonomic distribution of PKA catalytic and regulatory subunits used in phylogenetic analysis. Sequences provided in Supplementary Data.

Phylum	Number of Species	PKA Catalytic	PKA Regulatory		Total
			Type I	Type II	
Annelida	3	2	1	2	5
Arthropoda	150	227	191	148	566
Order Decapoda	59	131	105	97	333
Chordata	286	327	334	341	1002
Cnidaria	16	16	14	12	42
Echinodermata	14	16	9	11	36
Mollusca	17	26	14	17	57
Nematoda	6	7	3	0	10
Platyhelminthes	6	10	1	8	19
Porifera	6	6	4	5	15
Tardigrada	2	2	3	4	9

**Table 2:** Percent identity and similarity between human, fruit fly, and decapod PKA catalytic subunits. Percent identity is represented in the top quadrant above identical sequences, and percent similarity (*italics*) is represented in the lower quadrant below identical sequences. Shading is based on the range of identity or similarity from green (most similar) to orange (least similar).

			Chordate			Arthropod							
						Decapod							
			***H. sapiens* PKACα** NP_002721.1	***H. sapiens* PKACβ** P22694.2	***D. melanogaster* PKA-C_1_** DromelEVm006045t1/NP_476977.1	***G. lateralis* PKA-C_1_** GeclaM_EVm007738t2	***E. sinensis* PKA-C_1_** Erisi1_EVm008591t1	***C. maenas* PKA-C_D1_** CarmaC_EVm006935t3	***C. maenas* PKA-C_GLY2_** CarmaY_Evm006323t1	***C. quadricarinatus* PKA-C_1_** ChequaEVm003405t5	***P. vannamei* PKA-C_1_** PenvanEVm002816t3	***P. vannamei* PKA-C_GLY1_** XP_070000929.1	
**Chordate**		***H. sapiens* PKACα** NP_002721.1	100	92.9	82.4	86.9	87.2	81.7	62.1	86.3	81.3	59.2	Percent Identity
		***H. sapiens* PKACβ** P22694.2	*95.2*	100	82.2	86.0	86.3	80.4	61.2	86.0	80.2	58.1	
		***D. melanogaster* PKA-C_1_** DromelEVm006045t1/NP_476977.1	*89.0*	*89.2*	100	90.4	90.9	84.6	64.7	90.1	83.1	60.2	
**Arthropod**	**Decapod**	***G. lateralis* PKA-C_1_** GeclaM_EVm007738t2	*91.2*	*90.9*	*94.3*	100	99.2	91.3	69.4	99.2	89.8	64.1	
		***E. sinensis* PKA-C_1_** Erisi1_EVm008591t1	*91.5*	*91.2*	*94.6*	*99.7*	100	92.1	70.0	98.6	89.2	*63.6*	
		***C. maenas* PKA-C_D1_** CarmaC_EVm006935t3	*86.7*	*85.6*	*88.6*	*92.9*	*93.2*	100	71.3	90.7	81.8	62.0	
		***C. maenas* PKA-C_GLY2_** CarmaY_Evm006323t1	*65.6*	*64.8*	*68.1*	*70.9*	*71.1*	72.0	100	62.2	62.2	63.2	
		***C. quadricarinatus* PKA-C_1_** ChequaEVm003405t5	*91.2*	*91.5*	*94.6*	*99.4*	*99.7*	*92.9*	*66.4*	100	90.6	64.7	
		***P. vannamei* PKA-C_1_** PenvanEVm002816t3	*87.5*	*86.7*	*88.7*	*93.2*	*93.5*	*87.0*	*66.4*	93.8	100	71.7	
		***P. vannamei* PKA-C_GLY1_** XP_070000929.1	*64.3*	*63.2*	*65.1*	*67.0*	*67.2*	*65.6*	*68.5*	*67.4*	*72.1*	100	
			*Percent Similarity*										

**Table 3:** Percent identity and similarity between human, fruit fly, and decapod PKA regulatory type I subunits. Percent identity is represented in the top quadrant above identical sequences, and percent similarity (*italics*) is represented in the lower quadrant below identical sequences. Shading is based on the range of identity or similarity from green (most similar) to orange (least similar).

			Chordate			Arthropod									
						Decapod									
			***H. sapiens* PKA-RIα** NP_002725.1	***H. sapiens* PKA-RIβ** NP_001158232.1	***D. melanogaster* PKA-RI_AA_** DromelEVm005673t1/NP_001189147.1	***C. maenas* PKA-RI_1_** CarmaY_EVm009321t1	***C. maenas* PKA-RI_D1_** CarmaY_EVm009321t2	***E. sinensis* PKA-RI_1_** Erisi1_EVm007953t3/XP_050718363.1	***E. sinensis* PKA-RI_D1_** Erisi1_EVm007953t2	***G. lateralis* PKA-RI_D1_** GeclaM_EVm010036t1	***C. quadricarinatus* PKA-RI_1_** ChequaEVm006131t1	***C. quadricarinatus* PKA-RI_D1_** XP_053629376.1	***P. vannamei* PKA-RI_1_** PenvanEVm004491t1/ **XP_069981575.1**	***P. vannamei* PKA-RI_D1_** XP_069981583.1	
**Chordate**		***H. sapiens* PKA-RIα** NP_002725.1	100	81.4	70.4	67.5	58.5	68.0	59.3	58.3	67.5	58.8	66.8	59.8	Percent Identity
		***H. sapiens* PKA-RIβ** NP_001158232.1	*89.5*	100	71.5	69.0	59.8	70.1	60.6	59.8	69.0	60.1	67.6	61.2	
		***D. melanogaster* PKA-RI_AA_** DromelEVm005673t1/NP_001189147.1	*80.1*	*80.4*	100	73.7	63.7	74.8	64.7	61.8	73.7	63.9	71.1	64.5	
**Arthropod**	**Decapod**	***C. maenas* PKA-RI_1_** CarmaY_EVm009321t1	*79.8*	*80.3*	*83.6*	100	85.2	96.5	82.3	76.6	93.8	79.3	90.7	80.1	
		***C. maenas* PKA-RI_D1_** CarmaY_EVm009321t2	*68.8*	*69.3*	*72.7*	*85.5*	100	82.3	96.6	88.0	79.3	93.2	77.0	94.1	
		***E. sinensis* PKA-RI_1_** Erisi1_EVm007953t3/XP_050718363.1	*79.0*	*80.6*	*84.1*	*98.1*	*84.1*	100	85.2	79.0	95.2	81.2	91.0	80.9	
		***E. sinensis* PKA-RI_D1_** Erisi1_EVm007953t2	*68.2*	*69.3*	*73.2*	*84.1*	*98.5*	*85.5*	100	90.7	81.2	95.4	77.8	95.1	
		***G. lateralis* PKA-RI_D1_** GeclaM_EVm010036t1	*66.1*	*66.7*	*68.7*	*78.5*	*90.1*	*79.3*	*91.1*	100	76.3	87.7	72.4	86.4	
		***C. quadricarinatus* PKA-RI_1_** ChequaEVm006131t1	*79.3*	*80.3*	*84.1*	*96.5*	*82.0*	*96.8*	*82.8*	*77.2*	100	85.2	91.5	*80.7*	
		***C. quadricarinatus* PKA-RI_D1_** XP_053629376.1	*68.2*	*69.3*	*73.2*	*82*	*96.0*	*82.8*	*96.9*	*88.6*	85.5	100	80.7	*94.8*	
		***P. vannamei* PKA-RI_1_** PenvanEVm004491t1/ XP_069981575.1	*77.8*	*79.1*	*81.1*	*93.5*	*79.6*	*93.0*	*79.6*	*74.2*	*94.1*	*83.3*	100	81.9	
		***P. vannamei* PKA-RI_D1_** XP_069981583.1	*69.3*	*70.3*	*73.5*	*82.8*	*96.9*	*82.8*	*96.9*	*88.6*	83.3	97.5	*82.2*	100	
			*Percent Similarity*												

**Table 4:** Percent identity and similarity between human, fruit fly, and decapod PKA regulatory type II subunits. Percent identity is represented in the top quadrant above identical sequences, and percent similarity (*italics*) is represented in the lower quadrant below identical sequences. Shading is based on the range of identity or similarity from green (most similar) to orange (least similar).

			Chordate			Arthropod							
						Decapod							
			***H. sapiens* PKA-RIIα** NP_004148.1	***H. sapiens* PKA-RIIβ** NP_002727.2	***D. melanogaster* PKA-RII_A_** DromelEVm005674t1/NP_523671.1	***C. maenas* PKA-RII_GLY_** CarmaC_EVm008235t2	***G. lateralis* PKA-RII_GLY_** GeclaM_EVm006874t1	***G. lateralis* PKA-RII_D1_** GeclaM_EVm006874t3	***C. quadricarinatus* PKA-RII_GLY_** ChequaEVm005536t2	***C. quadricarinatus* PKA-RII_D1_** ChequaEVm005536t4	***P. vannamei* PKA-RII_GLY_** XP_069989562.1	***P. vannamei* PKA-RII_D1_** XP_069989583.1	
**Chordate**		***H. sapiens* PKA-RIIα** NP_004148.1	100	65.5	46.5	48.5	45.8	45.5	49.4	45.3	48.8	46.3	Percent Identity
		***H. sapiens* PKA-RIIβ** NP_002727.2	*76.4*	100	41.3	46.7	43.5	43.6	49.3	44.8	51.1	46.7	
		***D. melanogaster* PKA-RII_A_** DromelEVm005674t1/NP_523671.1	*60.5*	*58.9*	100	55.6	52.9	54.4	58.7	55.2	58.8	56.0	
**Arthropod**	**Decapod**	***C. maenas* PKA-RII_GLY_** CarmaC_EVm008235t2	*63.2*	*61.8*	*70.8*	100	70.3	72.4	79.7	67.8	79.2	69.0	
		***G. lateralis* PKA-RII_GLY_** GeclaM_EVm006874t1	*57.6*	*59.9*	*66.8*	*79.0*	100	77.9	70.7	67.2	71.2	67.2	
		***G. lateralis* PKA-RII_D1_** GeclaM_EVm006874t3	*57.3*	*57.9*	*66.2*	*76.2*	*78.6*	100	71.5	83.0	72.0	82.9	
		***C. quadricarinatus* PKA-RII_GLY_** ChequaEVm005536t2	*64.2*	*62.9*	*72.2*	*86.7*	*79.2*	*77.2*	100	81.6	89.9	74.9	
		***C. quadricarinatus* PKA-RII_D1_** ChequaEVm005536t4	*57.6*	*58.1*	*67.2*	*74.3*	*73.2*	*90.2*	82.4	100	74.6	86.2	
		***P. vannamei* PKA-RII_GLY_** XP_069989562.1	*62.6*	*63.2*	*72.9*	*85.7*	*78.5*	*76.9*	*93*	*77.7*	100	82.3	
		***P. vannamei* PKA-RII_D1_** XP_069989583.1	*58.4*	*58.6*	*69.3*	*74.3*	*72.3*	*89.7*	*78.2*	*90.6*	*83.1*	100	
			*Percent Similarity*										

**Table 5:** Taxonomic distribution of PKC sequences by subfamily used in phylogenetic analysis. Sequences provided in Supplementary Data.

Phylum	Number of Species	PKN	Conventional	Novel δ/θ	Novel ε/η	Atypical	Total
Annelida	5	2	3	1	2	1	9
Arthropoda	173	115	153	106	100	204	678
Order Decapoda	52	77	62	34	44	98	315
Chordata	231	132	223	173	185	147	860
Cnidaria	16	8	11	10	7	13	49
Echinodermata	17	17	8	12	12	16	65
Mollusca	23	11	14	26	12	26	89
Nematoda	10	4	4	3	3	3	17
Platyhelminthes	12	3	11	0	9	7	30
Porifera	7	6	9	7	9	4	35
Tardigrada	3	2	3	0	3	3	11

**Table 6:** Percent identity between human, fruit fly, and decapod PKC subfamilies. Human PKC subfamilies and isoforms (columns) and percent identity with corresponding fruit fly or decapod sequences are indicated for each isoform, using the proposed classification for decapod sequences. Shading is based on the range of identity within each subfamily from green (most similar) to orange (least similar). Asterisk (*) indicates an incomplete sequence.

	Conventional		Novel δ/θ		Novel ε/η		Atypical		PKN		
Human:	PKCα	PKCβ	PKCδ	PKCθ	PKCε	PKCη	PKCι	PKCζ	PKN1	PKN2	PKN3
*Drosophila melanogaster*	DromelEVm002580t1 PKC53E_F_		DromelEVm001975t2		DromelEVm001975t1 D1		DromelEVm002874t2		NP_788290.1		
	66.1	66.2	34.5	34.8	57.1	51.5	58.3	55.7	43.4	46.8	37.6
			DromelEVm002394t1				DromelEVm002874t1 PKC_AC_				
			45.8	40.3			65.1	61.0			
*Carcinus maenas*	CarmaY_EVm003058t1 cPKC_D1_		CarmaC_EVm004306t1 nPKC_D1δ_		CarmaY_EVm002701t1 nPKC_D1ε_		CarmaY_EVm004113t1 aPKC_D1_		CarmaY_EVm003480t1 * PNK_D1_		
	64.6	63.2	40.0	41.5	57.7	52.8	66.2	61.9	33.5	32.5	31.2
							CarmaY_EVm004113t2 aPKC_D2_				
							58.3	56.6			
*Gecarcinus lateralis*	GeclaM_EVm002835t1 cPKC_D1_		GeclaM_EVm003046t1 nPKC_D1δ_		GeclaM_EVm002409t1 nPKC_D1ε_		GeclaM_EVm003606t1 aPKC_D1_		GeclaM_EVm004548t1 * PNK_D1_		
	66.0	64.7	42.6	42.9	57.8	52.6	65.7	61.8	31.2	27.4	27.4
			GeclaM_EVm003046t2 nPKC_D1δ_								
			40.4	41.0							
*Eriocheir sinensis*	Erisi1_EVm005209t1 * cPKC_D1_		Erisi1_EVm001544t1 * nPKC_D1δ_		Erisi1_EVm002283t1 nPKC_D1ε_		Erisi1_EVm009429t2 * aPKC_D1_		Erisi1_EVm001069t3 PKN_D1_		
	49.1	49.2	35.9	36.2	57.9	52.5	43.0	41.6	47.2	51.4	42.6
*Cherax quadricarinatus*	ChequaEVm002328t1 cPKC_D1_		ChequaEVm001435t3 nPKC_D1δ_		ChequaEVm001960t1 nPKC_D1ε_		ChequaEVm002958t1 aPKC_D1_		ChequaEVm000705t3 PKN_D1_		
	58.0	51.7	42.7	40.0	58.0	51.3	66.4	61.8	44.4	47.9	39.7
									ChequaEVm000705t1 PKN_D2_		
	ChequaEVm002328t2 cPKC_D1_ ^Δ^						ChequaEVm002958t2 aPKC_D3_		42.3	46.4	37.7
	66.4	67.1					65.2	55.9	XP_053646711.1 PKND3		
									38.2	40.1	33.7
*Penaeus* (*Litopenaeus*) *vannamei*	PenvanEVm004159t1 cPKC_D1_		PenvanEVm003492t1 * nPKC_D1δ_		PenvanEVm001947t1 * nPKC_D1ε_		PenvanEVm001352t1 * aPKC_D1_		PenvanEVm000367t1 PKN_D1_		
	47.0	65.1	31.7	30.1	50.0	43.8	66.1	45.2	45.1	48.3	40.5

^Δ^ Sequence is identical to ChequaEVm002328t1 until the last 48 residues of the C-terminus.

**Table 7:** Proposed classification of Decapod PKA and PKC protein sequences. Sequences in selected decapod species: *Carcinus maenas*, *Gecarcinus lateralis*, *Eriocheir sinensis*, *Cherax quadricarinatus*, and *Penaeus* (*Litopenaeus*) *vannamei*, and their proposed classification. NCBI accession numbers are indicated in parentheses where available. Sequences are available in the Supplementary Data. Motif indicates the consensus sequence in the N-terminus that distinguishes decapod types. Asterisk (*) indicates an incomplete sequence.

		*C. maenas*	*G. lateralis*	*E. sinensis*	*C. quadricarinatus*	*P. vannamei*	Proposed Classification	Motif
**PKA**	**Catalytic**		GeclaM_EVm007738t2 (PX409858)	Erisi1_EVm008591t1	ChequaEVm003405t5	PenvanEVm002816t3	PKA-C_1_	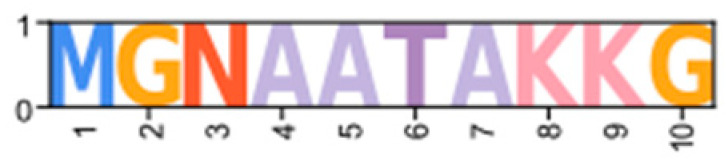
				XP_050715590.1	ChequaEVm003405t4	XP_070000929.1	PKA-C_GLY1_	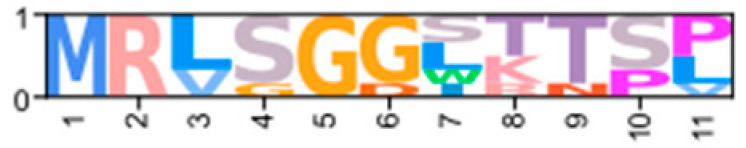
		CarmaY_Evm006323t1 (PX409857)			ChequaEVm003405t3		PKA-C_GLY2_	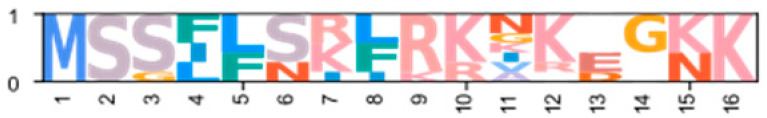
		CarmaC_EVm006935t3	GeclaM_EVm007738t1	XP_050715595.1			PKA-C_D1_	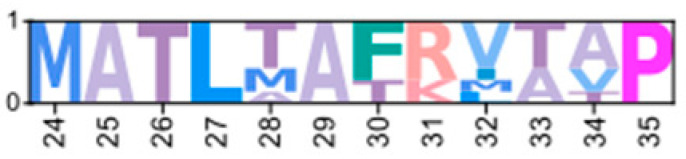
	**Regulatory I**	CarmaY_EVm009321t1 (PX409859)		Erisi1_EVm007953t3 (XP_050718363.1)	ChequaEVm006131t1	PenvanEVm004491t1 (XP_069981575.1)	PKA-RI_1_	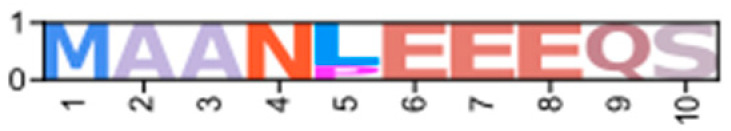
		CarmaY_EVm009321t2 (PX409860)	GeclaM_EVm010036t1 (PX409861)	Erisi1_EVm007953t2	XP_053629376.1	XP_069981583.1	PKA-RI_D1_	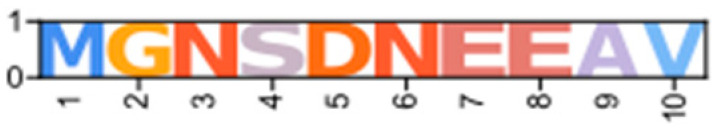
	**Regulatory II**	CarmaC_EVm008235t2 (PX409862)	GeclaM_EVm006874t1 (PX409863)		ChequaEVm005536t2	XP_069989562.1	PKA-RII_GLY_	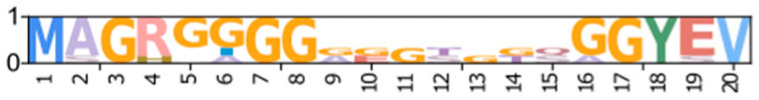
			GeclaM_EVm006874t3		ChequaEVm005536t4	XP_069989583.1	PKA-RII_D1_	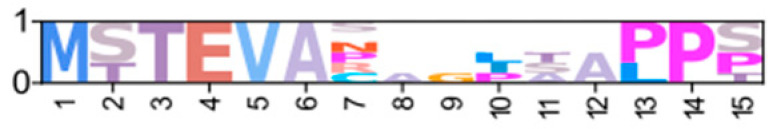
**PKC**	**PKN**	CarmaY_EVm003480t1 *	GeclaM_EVm004548t1 *	Erisi1_EVm001069t3 (XP_050729390.1 *)	ChequaEVm000705t3	PenvanEVm000367t1	PKN_D1_	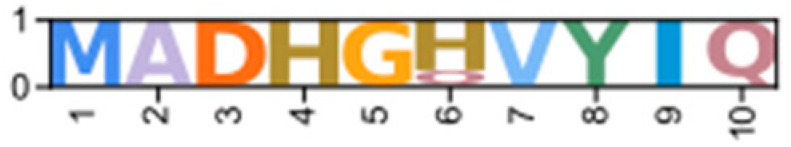
	**Conventional**	CarmaY_EVm003058t1 (PX409868)	GeclaM_EVm002835t1 (PX409871)	Erisi1_EVm005209t1 *	ChequaEVm002328t1	PenvanEVm001352t1	cPKC_D1_	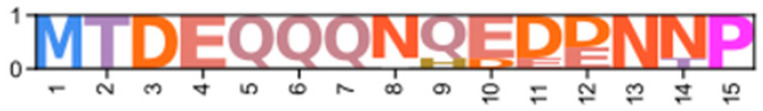
	**Novel δ**	CarmaC_EVm004306t1 (PX409866)	GeclaM_EVm003046t2 (PX409874)	Erisi1_EVm001544t1 *	ChequaEVm001435t3	PenvanEVm003492t1 *	nPKC_D1δ_	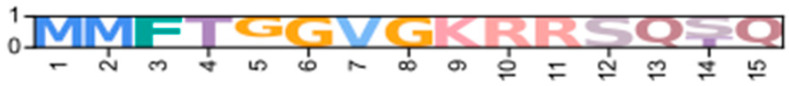
	**Novel ε**	CarmaY_EVm002701t1 (PX409865)	GeclaM_EVm002409t1 (PX409873)	Erisi1_EVm002283t1	ChequaEVm001960t1	PenvanEVm001947t1 *	nPKC_D1ε_	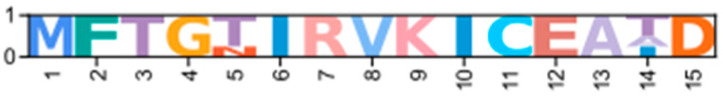
	**Atypical**	CarmaY_EVm004113t1 (PX409869)	GeclaM_EVm003606t1 (PX409872)	Erisi1_EVm009429t2 *	ChequaEVm002958t1 (XP_069952440.1)	PenvanEVm004159t1 *	aPKC_D1_	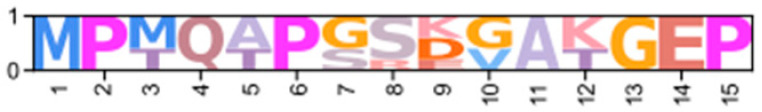

## Data Availability

Fasta files of all sequences and alignments presented in this study, tree files, and an Excel spreadsheet of sequence metadata are available in the Supplementary Data. PhyloPic credits for images used in phylogenetic trees are available in the Supplementary Text. Further inquiries can be directed to the corresponding authors.

## Supplementary Materials

The following supporting information can be downloaded at https://www.mdpi.com/article/10.3390/ijms262110585/s1.
